# Initial Evaluation of an Electronic Symptom Diary for Adolescents with Cancer

**DOI:** 10.2196/resprot.2175

**Published:** 2012-12-11

**Authors:** Christina Baggott, Faith Gibson, Beatriz Coll, Richard Kletter, Paul Zeltzer, Christine Miaskowski

**Affiliations:** 1 University of California, San FranciscoDepartment of Physiological NursingSan Francisco, CAUnited States; 2 London South Bank UniversityDepartment of Children's Nursing Health & Social CareLondonUnited Kingdom; 3 Great Ormond Street Hospital for ChildrenLondonUnited Kingdom; 4 San Francisco Unified School DistrictSan Francisco, CAUnited States; 5 GoMed SolutionsLos Angeles, CAUnited States

**Keywords:** mHealth, eHealth, patient-reported outcomes, symptom assessment, adolescent, cancer

## Abstract

**Background:**

The delivery of optimal care depends on accurate communication between patients and clinicians regarding untoward symptoms. Documentation of patients’ symptoms necessitates reliance on memory, which is often imprecise. We developed an electronic diary (eDiary) for adolescents and young adults (AYAs) with cancer to record symptoms.

**Objective:**

The purpose of this paper is to describe the utility of an eDiary designed for AYAs with cancer, including dependability of the mobile application, the reasons for any missing recorded data, patients’ adherence rates to daily symptom queries, and patients’ perceptions of the usefulness and acceptability of symptom data collection via mobile phones.

**Methods:**

Our team developed an electronic symptom diary based on interviews conducted with AYAs with cancer and their clinicians. This diary included daily severity ratings of pain, nausea, vomiting, fatigue, and sleep. The occurrence of other selected physical sequelae was assessed daily. Additionally, patients selected descriptors of their mood. A 3-week trial of the eDiary was conducted with 10 AYA cancer patients. Mobile phones with service plans were loaned to patients who were instructed to report their symptoms daily. Patients completed a brief questionnaire and were interviewed to elicit their perceptions of the eDiary and any technical difficulties encountered.

**Results:**

Overall adherence to daily symptom reports exceeded 90%. Young people experienced few technical difficulties and reported benefit from daily symptom reports. Symptom occurrence rates were high and considerable inter- and intra-patient variability was noted in symptom and mood reports.

**Conclusions:**

We demonstrated the utility of an eDiary that may contribute insight into patients’ symptom patterns to promote effective symptom management.

## Introduction

Adolescents and young adults (AYAs) with cancer experience numerous sequelae during treatment [1-5], which are associated with decrements in their quality of life [6]. Accurate communication between young people and their clinicians about these symptoms is crucial for the delivery of optimal supportive care. Furthermore, accurate symptom assessment during clinical trials is needed to advance the science of supportive care in oncology.

Current practices to determine symptom occurrence in clinical trials require that patients accurately recall their symptoms during clinic visits over the prior 1 to 6 weeks and report them to their clinicians. Clinicians then record this information in the medical record and researchers abstract the information to a database for analysis. Omissions or errors at any of these steps result in an underreporting or misrepresentation of symptom occurrence. Moreover, recent data suggests that the accuracy of symptom appraisal and recall depends on the use of short recall periods [7].

Alternatively, symptom diaries in paper format that are completed daily can be used to circumvent the challenges described above. However, patient adherence with these diaries is low, ranging from 11% to 56% [8-12]. In addition, adherence with paper diary entries is markedly low when real time data entry is checked electronically (eg, with light sensors to determine when the diary was opened) against the paper submission. In one study, 90% (approximately 36/40) of patients reported temporal adherence with paper diary entries. However, the true adherence level was 11% when the actual time that diary entries were written was monitored electronically. Many patients recorded entries just prior to returning the diary, known as “parking lot entries” [11]. Data based on inaccurate symptom recall may lead to inappropriate conclusions when symptom management studies are based on poorly recalled data [13].

The collection of AYAs’ symptom data in real-time using electronic diaries (eDiaries) could avoid poorly recalled data. Patients’ and clinicians’ interests in using eDiaries are high [14, 15]. The portability of these devices and availability of audible prompts may promote adherence with data entry. In a review of 62 studies that used electronic pain diaries [15], the overall adherence with diary entries was 83%. Factors that promoted adherence to eDiaries included the following: shorter diaries, having a user’s manual, financial compensation, and reminder alarms [15].

While initial eDiary research involved the use of personal digital assistants [14, 16-20], mobile phones are now also used for collecting a variety of data. Typically, the frequency of symptom assessment using eDiaries ranges from 1 to 12 times per day [21]. Entries can be triggered by random alerts, symptom events, or at predetermined schedules. Mobile phone diaries have been used by child and adult patients to monitor symptoms, alert clinicians about severe symptoms [22], or to deliver self-care interventions based on symptom assessments [23]. The term ecological momentary assessment (EMA) is used to describe the collection of real-time data from individuals in naturalistic settings. In pediatrics, EMA has been instrumental in the study of physical activity [24-30], affective mood disorders [31-34], evaluation of alcohol and illicit drug use [35], and smoking cessation [36]. These studies included multiple evaluations per day, as frequent as every 15 minutes, among relatively healthy individuals. No such studies were conducted with hospitalized AYAs.

AYAs with cancer have unique needs related to frequent and complex exacerbations of their disease- and treatment-related symptoms that could affect their abilities to maintain eDiaries. The lack of an eDiary geared to the symptoms and developmental needs of AYAs with cancer prompted us to develop a mobile application, titled the Mobile Oncology Symptom Tracker (mOST). To do so, we assembled a multidisciplinary team of 2 nurses and 3 software developers from GoMed Solutions, one of whom is a pediatric oncologist. We elicited input from 15 AYA oncology patients from two University-affiliated children’s hospitals in the inpatient or clinic settings. Semi-structured interviews were used to determine their use of technology, and ideas on the types of symptoms to monitor, frequency of reporting, and preferences for icons and graphics. In addition, clinicians and research experts were consulted during the development of the application. The symptoms selected for daily monitoring were those that occur frequently during treatment of cancer [4]. Assessment of these symptoms was done using modifications of valid and reliable instruments when possible (Multimedia Appendix 1). Considering the complex demands placed on patients undergoing cancer therapy, we designed the application to collect symptom data as a single end-of-day (EOD) entry to minimize patient burden. This single entry aims to capture the majority of the day’s ratings, and the daily component circumvents issues related to poor recall associated with entries that are made on subsequent days. Entries could be made between 3 pm and midnight at the convenience of the patient. A team of researchers and developers used the application for 2 weeks to evaluate its functionality prior to clinical testing.

The purpose of this paper is to describe the utility of mOST tested on a group of 10 oncology patients, ages 13 to 21. The specific aims of this pilot study were to determine the dependability of the mobile application, the reasons for any missing entries and patients’ adherence rates to daily symptom queries for multiple symptom reporting. In addition, patients’ perceptions of the usefulness and acceptability of symptom data collection via mobile phones were evaluated after a 3-week trial assessment period.

## Methods

### Participants

In this descriptive, longitudinal study, we evaluated a mobile phone-based electronic symptom diary in a convenience sample of AYAs with cancer. Patients were eligible to participate if they were 13 to 21 years of age, could understand English, gave assent or consent to participate, had not participated in the prior application development interviews, and were receiving chemotherapy (for initial therapy, relapsed, or refractory disease). The study was approved by the Human Subjects Committee at the University of California, San Francisco.

Eligible patients were identified with assistance from pediatric oncology clinics and in-patient advanced practice nurses. Research staff approached these patients to determine interest in the study. If patients expressed interest, the research staff informed them of the study procedures, risks, and benefits. Patients 18 years of age or older and the parents/guardians of younger patients signed written, informed consent. Younger patients gave written assent. Between March and April, 2011, 11 out of 13 patients approached agreed to participate in the study (response rate of 85%). Patients who refused were too busy or too ill to participate.

### Procedures

Patients completed a demographic form that included their age, race/ethnicity, primary diagnosis, date of diagnosis, number of relapses, prior mobile phone use, and approximate household income. An 8 GB iPhone 3GS, charger, and earphones were loaned to each participant during the study. The monthly service plan included: 450 minutes of peak time voice minutes, and unlimited data, text messaging, and evenings/weekend calling. The mOST application was downloaded on each phone. Patients received an instructions booklet and a tutorial on the application and phone use, expectations of the frequency of symptom reporting, and methods to notify research staff of system malfunctions. They were instructed to report their symptoms at the end of the day. A single daily report was designed to minimize patient burden. Patients could only access the system between 3 pm and midnight, as reports earlier in the day might not accurately reflect all of their experiences for the day. They could program 2 reminder messages to input daily entries with audible alerts, customizable for text choice and time selection. Data were delivered to a secure website, listed by study number only, and could be downloaded to database and statistical packages for analysis. The dates and times of data submission and data upload were coded.

### Symptom Assessments

Severity ratings of 5 disease or treatment-related symptoms were assessed daily: pain, nausea, vomiting, fatigue, and sleep quality. A body diagram was used to indicate the location(s) of any pain experienced. A visual analog scale (VAS) was selected to assess pain intensity (ie, no pain or worst pain) based on a consensus statement compiled by pediatric pain researchers [37]. We selected the Color Analog Scale (CAS) as the VAS for this study due to its excellent psychometric properties [38, 39]. However, pain scales that include face icons (ie, faces pain scales) were preferred by pediatric and adolescent patients in a prior study [40] and by many of the AYAs we interviewed during the software development period. We included the Faces Pain Scale-Revised (FPS-R), a valid and reliable pediatric pain measure [41], as well as a VAS to assess pain, with the consideration that no additional burden was placed on participants. The FPS-R appeared as 6 faces on the bottom of the mOST screen. When a patient tapped a face, the selection enlarged and filled most of the screen. Patients then responded to the statement, “drag the slider to show your average pain level in your <body part> since midnight”, using the CAS.

Validity of the CAS was previously evaluated by eliciting pain ratings from 30 children in an emergency department (ED) who reported pain [39]. Initial median pain ratings were higher than those obtained after analgesic administration, 6 centimeter (cm) and 3.1 cm, respectively. Median scores of 30 children without pain were 0 cm, compared to median ratings of 7.0 cm from children who reported severe pain. In addition, correlations between CAS scores and the FPS-R were positive and strong (ie, 0.89) [39]. In another study, reliability of the CAS was evaluated by obtaining 2 pain ratings on the CAS from patients in a pediatric ED, 1 minute apart. The intraclass correlation coefficient in this study was high (*r*=0.97, 95% CI 0.95-0.98) [38]. Previously, strong positive correlations were noted between FPS-R scores and children’s pain ratings on a VAS. The validity of the FPS-R was evaluated by eliciting pain reports from children after routine ear piercing [41]. In this study, similar correlations were noted in hospitalized children who experienced painful conditions [41]. Additional reports of the psychometric properties of the FPS-R are summarized in a review by Tomlinson and colleagues [42]

To assess nausea, we included the Pediatric Nausea Assessment Tool (PeNAT), a valid and reliable instrument to assess nausea among pediatric oncology patients [43]. In previous evaluations, PeNAT scores varied significantly among children admitted for different therapies (ie, children with cancer admitted for routine chemotherapy, children receiving conditioning therapy for hematopoietic stem cell transplants, children with cancer admitted for febrile neutropenia, and children without cancer admitted to the general pediatrics unit). Moderate correlation was noted among children’s PeNAT scores and their emetic episodes as well as with parental reports of their nausea. Test-retest reliability, measured by the collection of patient entries 1 hour apart, revealed moderate correlations between the 2 entries [43]. This tool was selected for the eDiary, with faces depicted in a similar fashion as the FPS-R. We used the PeNAT in conjunction with a VAS to assess the degree of nausea in this early stage of application development to collect correlative assessment data and to explore the optimal assessment tool for the application.

No single-item validated instruments were available to assess vomiting, fatigue, or sleep among AYA patients. The number of vomiting episodes was assessed with the single forced-choice query of: none, 1 time, or 2 or more times. Fatigue was assessed with 2 queries from the Fatigue Scale-Adolescent [44] (“my body has felt tired” and “my mind has felt worn out”) using a VAS format to collect data on physical fatigue (not tired or most tired) and mental fatigue, (not worn out or most worn out) respectively. Sleep quality was assessed with a single query in a VAS format (terrible or great) in conjunction with patient estimates of bedtime and wake time. All VAS scales on the application were in CAS format and transformed to a 0-100 scale, with higher values indicating more severe symptoms.

The occurrence of diarrhea, constipation, fever, numbness/tingling, mouth sores, dizziness, and headaches were assessed daily. These symptoms were included due to their frequent occurrence among pediatric oncology patients and their variability during treatment [4]. Patients could select symptoms from a list and highlight the text by swiping the printed text with one finger from left to right or type in other conditions. In addition, patients selected descriptors for their current mood - the choices included were angry, scared, frustrated, lonely, anxious, irritable, worried, happy, confident, and hopeful. Both positive and negative terms to describe mood were selected for inclusion based on their frequent reports in clinical encounters.

Patients received $1 credit towards a department store gift card for each diary entry. In addition, those who completed ≥ 90% of the assessments in a 21-day period were given a $50 gift card. If patients attempted to report symptoms but experienced a system error, the attempt was also applied towards the gift card credits.

### Post Use Evaluation

The principal investigator or research assistant conducted 10 to 15 minute interviews with patients at the completion of their evaluation period to obtain their perceptions about using mOST and any technical difficulties they experienced. In addition, they were asked to carry out a “think-aloud” exercise [45] where they provided the rationales for the actions they made on mOST as they did them. These interviews were audio recorded and transcribed verbatim. All patient identifiers were removed from the transcripts. A brief questionnaire was completed at the time of the interview to elicit patients’ experiences with the application.

### Data Analysis

Descriptive statistics were used to characterize demographic and clinical characteristics as well as rates of symptom occurrence. The total number of system errors was calculated during the 21-day study period. Data entry adherence rates were determined by calculating the number of symptom reports over the 21 days of the study. One-way analysis of variance was used to evaluate the differences in patients’ weekly adherence rates. The interview transcripts were analyzed by 2 researchers, with verification by 1 of the senior investigators.

## Results

### Patient Characteristics

The demographic and clinical characteristics of the AYAs who participated are described in Table 1. A patient completed 2 entries just prior to being notified that her tumor had progressed. Because she required emergency surgery followed by a transfer to the pediatric intensive care unit (PICU), she was withdrawn from the study. Another patient completed 13 daily entries prior transfer to the PICU due to complications following hematopoietic stem cell transplantation. Her symptom reports are included in this analysis but she was not interviewed. All study phones were returned at the completion of the study, but several had misplaced the phone chargers or earphones.

The patients’ baseline technology use is described in Table 2. All but 2 (a 15 year old male and a 20 year old male who was homeless), of the AYAs owned a mobile phone with service plans primarily paid for by their parents. Daily use of the phones for voice calls and text messaging was common but not uniform. All patients owned a computer and only 1 did not have home Internet access. Most patients (80 %) had a social networking account.

**Table 1:** Demographic and clinical characteristics of participants (N=10).

Characteristic^a,b^		n (%)
**Gender**		
	Male	6 (60%)
	Female	4 (40%)
**Race/Ethnicity**		
	Hispanic White	3 (30%)
	Non-Hispanic White	1 (10%)
	African American	1 (10%)
	Hispanic-Other or not specified	5 (50%)
**Diagnosis**		
	Leukemia/Lymphoma	6 (60%)
	Bone tumor	3 (30%)
	Sarcoma-other	1 (10%)
**Number of prior relapses**		
	None	5 (50%)
	One or more	5 (50%)
**Setting**		
	Inpatient	4 (40%)
	Outpatient	4 (40%)
	Both	2 (20%)

^a^Age in years (SD) = 18.2 (2.9)

^b^Time since initial diagnosis in months (SD)= 12.2 (15.3)

**Table 2:** Technology use among participants.

Question	Response	n (%)
Do you have a mobile phone?	Yes	8 (80%)
	No	2 (20%)
Who pays for the service?	Self	2 (20%)
	Parents	6 (60%)
	Not applicable	2 (20%)
How often do you use your phone for voice calls?	Daily	4 (40%)
	4-6 days/week	2 (20%)
	2-3 days/week	2 (20%)
	Not applicable	2 (20%)
How often do you use your phone for text messaging?	Daily	5 (50%)
	4-6 days/week	2 (20%)
	2-3 days/week	1 (10%)
	Not applicable	2 (20%)
Do you have a computer at home?	Yes	10 (100%)
	No	0 (0%)
Do you have Internet access at home?	Yes	9 (90%)
	No	1 (10%)
Is Internet access WiFi enabled?	Yes	5 (50%)
	No	2 (20%)
	Not applicable	1 (10%)
	Not sure	2 (20%)
Do you have a page on Facebook or MySpace?	Yes	8 (80%)
	No	2 (20%)

### Dependability of the Mobile Application

On day 14 of our data collection period, we noted a system malfunction that occurred if patients skipped a day of diary entries. On the subsequent day, the application did not function. During the “beta-testing” period, testers skipped data entries and did not experience this malfunction. An interim solution was devised in which patients deleted the application and reloaded it from a website. Subsequently, we monitored the database daily. If patients missed an entry, we contacted them and provided instructions on how to reload the application. A total of 3 episodes of system malfunction occurred over the study period. The software malfunction was rectified by the end of the study. An error message appeared at the time of upload for 3 patients, but that the entry was transmitted the next time the application was opened.

### Reasons for Missing Data

One patient missed an eDiary entry when he felt too ill to enter data. He missed the subsequent day due to the system malfunction and a third day when he went out of town and left the phone at home. Another patient missed a data entry on a day he discovered that his leukemia had relapsed. He missed 4 consecutive entries during a period when we attempted to reach him. He then reloaded the application and maintained full adherence until the end of his study period. A third patient missed 2 entries due to forgetfulness. A 4^th^ patient reported full adherence to the data entries, but we noted missing data on 1 day with 2 entries the subsequent day, all uploaded on the third day. This finding likely represented Internet access difficulties, but we coded her data as missing a day to be conservative in adherence estimations.

### Adherence

Adherence can be calculated by assessing different causation end points. When data were counted as missing only for omitted entries due to forgetfulness and illness-related concerns of mild to moderate levels (ie, giving credit for days of system error and time spent in the PICU), the overall adherence rate for daily symptom reports during the 21 day study was 97%. When days of system malfunction were included as missing data, the adherence rate was 95%. When all days of missing data were used in the calculation, including the 8 days when a patient spent was in the PICU, the adherence rate was 91%. Adherence rates did not vary among the 3 weeks (F_2,27_=1.016, *P* =.38).

### Usefulness

**Figure 1:**
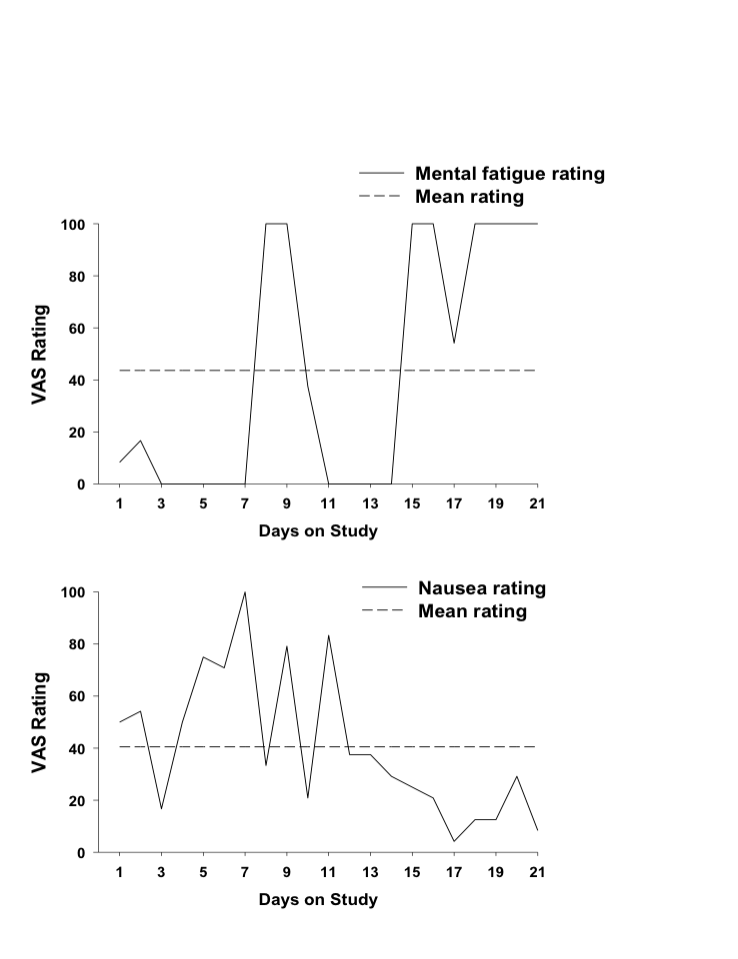
Mental fatigue and nausea ratings for participant 5. Mental fatigue rating in response to the statement “My mind has felt worn out” on a 0-100 VAS, where higher values indicate more severe fatigue. Nausea rating is response to nausea query on a 0-100 VAS, where higher values indicate more severe nausea. Dashed line represents mean values over the 21-day study period.

The data collected were used to delineate the potential utility of the eDiary to clinicians and researchers. Summaries of the symptoms and moods reported by individual patients are listed in Tables 3 and 4, respectively. Additional physical symptoms typed in and added by patients were: jaw pain, numb chin, bloody stools, bone ache, lightheaded, tinnitus, stomachache, and itchiness. Two patients wrote in symptoms already evaluated on the application (ie, “fatigue, tiredness” and “queasy nauseous”). Additional mood descriptors reported by patients were: giggly, bored, overwhelmed, and blah. In addition, trajectories reported by one patient of the severity of mental fatigue and nausea over the 3-week course are depicted in Figure 1 along with his mean values for each symptom over the 21 day study period, as exemplars of symptom variability over time. Considerable inter-patient and intra-patient variability were noted in the symptom and mood reports. The ability to collect this variability in patients’ experiences supports the usefulness of the eDiary.

### Acceptability

In an exit interview, one patient with peripheral neuropathy noted that the highlighting feature was difficult to master. Others stated that the highlighting feature was simple to use. The body diagram used to describe pain locations was divided into 9 regions–head/neck, hands, arms, chest, abdomen, upper back, lower back/pelvis, legs, and feet. Difficulties with pinpointing the precise location of pain were mentioned by 4 patients (eg, the indicator to depict neck pain showed up on the head).

Patients rated the application as either “easy” (2/9, 22%) or “very easy” (7/9, 78%) to use. Daily entries were generally completed in less than 2 minutes. One patient had mild visual deficits and mentioned that the print was difficult to read at times. However, she was able to read the screens out loud during the “think-aloud” exercise. All other patients reported that the print size posed no difficulties. In the exit survey, patients were asked to select or write in adjectives to describe the application. They could select more than 1 descriptor. All of the selected words had a positive connotation (ie, 67% rewarding, 78% valuable, 78% educational, 67% interesting). No one selected challenging, frustrating, time consuming, worthless, confusing, or difficult to describe mOST. All patients who were questioned (n=7) reported that they would recommend the application to others. Selected quotations regarding why they would recommend the application to others are included in Table 5. Patients’ preferences for the use of the VAS or faces scales to rate pain and nausea were fairly evenly divided. Those who preferred the VAS appreciated the opportunity to select a more precise level of pain or nausea, compared to only a few options on the faces scales. Those who preferred the faces scales commented that the diagrams depicted how they were feeling.

**Table 3:** Patients’ reports of physical symptoms.

Symptom	Percentage of days each patient reported symptoms										Mean of symptom occurrence rates		% of patients reporting symptoms
	Patient												
	1	2	3	4	5	6	7	8	9	10			
	Inpatient				Both in and outpatient		Outpatient						
Fatigue-Physical^a^	100	100	100	86	43	21	90	62	39	5	64	100	
Fatigue-Mental^a^	95	92	88	81	48	16	90	52	44	5	62	100	
Nausea^b^	100	100	65	24	95	47	75	0	29	38	57	90	
Headache	95	54	29	81	29	0	5	52	22	76	44	90	
Pain^c^	81	69	65	43	38	0	10	0	24	33	36	80	
Dizzy	52	46	24	24	29	0	30	5	17	33	26	90	
Numb	62	8	100	5	0	100	0	0	0	5	10	60	
Constipation	29	0	65	0	10	0	55	0	11	0	10	50	
Diarrhea	29	92	6	5	5	0	0	0	0	0	14	50	
Mouth sores	0	39	0	5	0	0	5	19	39	0	11	50	
Fever	0	31	6	10	0	0	0	0	0	5	5	40	

^a^Occurrence rate based on percentage of days that VAS score >30 on 0-100 scale

^b^Occurrence rate based on percentage of days that patients selected face 2, 3, or 4 on the Pediatric Nausea Assessment Tool (PeNAT)

^c^Occurrence rate based on percentage of days that patients selected face 2, 3, 4, 5, or 6 on the Faces Pain Scale-Revised (FPS-R)

**Table 4:** Patients’ selection of mood descriptors.

Mood descriptor	Percentage of days each patient selected mood descriptors										Mean of mood occurrence rates	% of patients reporting mood
	Patient											
	1	2	3	4	5	6	7	8	9	10		
	Inpatient				Both in and outpatient		Outpatient					
**Positive mood descriptors**												
Hopeful	38	0	65	86	33	26	5	43	22	91	41	100
Happy	67	15	82	0	62	26	5	52	33	24	37	90
Confident	52	15	12	86	5	37	0	10	17	86	31	90
**Negative mood descriptors**												
Frustrated	62	23	53	10	43	16	10	0	33	86	34	90
Irritable	0	46	12	52	38	16	15	5	56	71	31	90
Worried	0	46	59	10	19	11	40	10	0	81	28	80
Anxious	38	8	65	0	19	5	10	10	11	95	26	90
Sad	0	46	47	57	10	11	0	5	6	33	21	80
Angry	19	0	24	5	14	5	0	0	11	48	13	80
Scared	0	31	24	0	5	0	0	5	0	24	9	50
Lonely	0	0	18	33	0	0	0	0	0	0	5	20

**Table 5:** Participants’ reasons for recommending the use of the mOST application to others.

Participant	Quotation
18 year old, Hispanic female	“…it's a good idea to keep like, to keep a list of how you're feeling"
20 year old Hispanic male	“…it kind of like channels whatever you have wrong with you like out…”
21 year old Hispanic male	“… because it really helps to see how you've been doing too actually, not just like, you know, go on day by day. But it does help you see and reflect on how you're doing. And if you did something the other day that helped, will help you make you feel better and so on. “
19 year old Hispanic female	“…because "It’s a good app and it just makes you -- I know for me it makes me feel better because I can keep track of my symptoms, kind of like a self-comforting type of thing because you know what you’re going through and you’re not scared. And eventually if this does go to where the doctors can see it, it’d be even better because you know that your physician is seeing it and the communication’s there.”

## Discussion

This study is the first to demonstrate that the use of an eDiary on a mobile phone platform is a feasible method to collect daily symptom data from AYAs with cancer. The adherence rates in this study (>91%) greatly exceed those associated with pen and paper diaries [8-12], and did not vary significantly over the 3-week period. Patients reported that the application was simple to use and that they would recommend its use to others. The daily collection of patients’ symptom experiences may be very useful in pediatric oncology, as it opens new doors to study symptom trajectories in this population. With this technology, researchers can evaluate daily symptom reports and potentially discover patterns that may lead to a better understanding of the etiology of various symptoms. This level of assessment of symptoms may increase our ability to identify those at highest risk for unrelieved symptoms and immediately intervene to relieve suffering.

Not all diseases or treatment-related symptoms experienced by cancer patients warrant daily monitoring. However, many symptoms are labile, particularly during chemotherapy [4]. Weekly or monthly averages of such symptoms provide an incomplete picture of the patient’s experience. Daily symptom reports provide clinicians with the information they need to intervene at an early stage and to encourage patients to monitor changes in symptom severity following an intervention. This variability cannot be appreciated when researchers report only group means. Mobile technology allows for the expeditious collection of daily symptom reports, avoiding the burden placed on AYAs when maintaining a paper diary.

Researchers can evaluate within-subject changes using the statistical process of multilevel modeling [46, 47]. In addition, with daily symptom reports, a more accurate evaluation of symptom clusters may be possible when temporal co-occurrence is confirmed. Through the use of an eDiary, one can evaluate changes in patients’ mood descriptors to determine the relationships among a variety of mood states and physical symptoms and timing of therapies.

Daily reports may not be sufficiently frequent among a group of critically ill patients. Broderick and colleagues [13] compared symptom data collected multiple times per day with EMA to subsequent patient recall with varying recall periods. Although patients had difficulties accurately recalling events more than 2 days in the past, EOD reports were deemed sufficient. The average of the day’s EMA reports strongly correlated with those recalled at the EOD [13]. However, the collection of EMA symptom ratings may be an advantageous option to evaluate symptom management strategies for some highly variable symptoms, such as novel treatments for nausea or pain.

EMA trials which involve frequent assessments throughout the day have been conducted among AYA patients with chronic pain [20], diabetes [48], and among healthy college students to assess smoking[ 49] and alcohol use [49]. However, to our knowledge, no studies have been done with AYA patients with cancer. One must weigh the potential for improved temporal resolution of symptom patterns with the potential for increased patient burden and subsequent decrements in adherence to monitoring. However, research on the effects of frequent monitoring on adherence with symptom reporting is lacking.

The use of mobile phones to collect symptom characteristics offers many advantages over pen-and-paper diaries for AYAs with cancer. AYAs are extremely comfortable with technology and may become more fully engaged in their care when they are empowered to track symptoms in a manner that resonates with them. AYA may prefer eDiaries to report sensitive information. In one study, adolescents reported more sexual behavior using a computer assessment compared with paper forms [50]. Thus, the use of electronic data collection may lead to more accurate and complete symptom profiles. Data collected electronically are date and time stamped to assure temporal accuracy. In contrast, reports of back filling (ie, entering data at a later date) and even forward filling (ie, entering data for future dates) of pen and paper diaries exist [51]. Electronic diaries place minimal burden on patients and clinicians can be alerted to serious concerns in real-time. In addition, eDiaries have the potential for seamless integration with web portals and electronic health records for in-depth evaluation and dissemination.

Currently our team is further evaluating the psychometrics of the mOST application. In this study, patients complete the eDiary daily over a 3-week course. On day 8 of the study they complete a series of symptom questionnaires and a quality of life measure (ie, the Pediatric Quality of Life Inventory) using a 1-week recall. Their daily eDiary responses during the first week will be compared to their recall of events reported on the symptom measures. Once the validity and reliability of the instrument are established, we plan to incorporate it into mHealth symptom intervention studies among AYA patients with cancer.

Although the use of mobile phones in research is considered an expensive endeavor, older models of phones are often available free of charge or for nominal fees. Mobile phone use among young people has been prevalent for many years and the age for acquiring smartphones is rapidly decreasing [52]. Mobile technology developers are often able to design applications that are device and platform agnostic and can be used on most phones. Thus researchers and clinicians can offer symptom assessment tools for use on patients’ own devices for very little cost. In addition, direct data entry from mobile devices eliminates the potential costs of data entry and validation by staff.

The limitations of this study need to be acknowledged. These limitations include the use of a convenience sample of only 10 patients and a relatively short trial period of 21 days. The patients received a relatively high incentive of up to $71 for their participation in the study, an acceptable practice in the United States considering the patients’ 3-week commitment to the study, but not standard in all countries [53]. In 2002, Institutional Review Board members were surveyed to determine their recommendations for pediatric incentives in research. The maximum allowed payment to children varied widely from $10-$1000, with a median of $100 [54]. The incentives in our study may have promoted improved adherence with daily reporting. The evaluation of the impact of study incentives on patient adherence is an appropriate subject for future investigation, along with the investigation of non-monetary interventions to promote prolonged engagement with technology. Despite these limitations, to our knowledge, this study is the first evaluation of an eDiary on mobile phones for AYAs with cancer. The 21^st^ century is an age in which technology is expected to revolutionize the collection of patient-reported outcomes and that may lead to important improvements in symptom management.

## Multimedia Appendix 1

mOST screen shots.

## Abbreviations

AYA: adolescent and young adult
CAS: color analog scale
ED: emergency department
eDiary: electronic diary
EMA: ecological momentary assessment
EOD: end-of-day
FPS-R: faces pain scale-revised
mOST: mobile oncology symptom tracker
PeNAT: pediatric nausea assessment tool
VAS: visual analog scale
