# Supplementary material for: Initial Evaluation of an Electronic Symptom Diary for Adolescents with Cancer
Source: JMIR Res Protoc. 2012 Dec 11;1(2):e23. doi: 10.2196/resprot.2175 (PMC3626160; doi:10.2196/resprot.2175)
Supplement: Supplementary file 1 [file resprot_v1i2e23_app1.pdf]

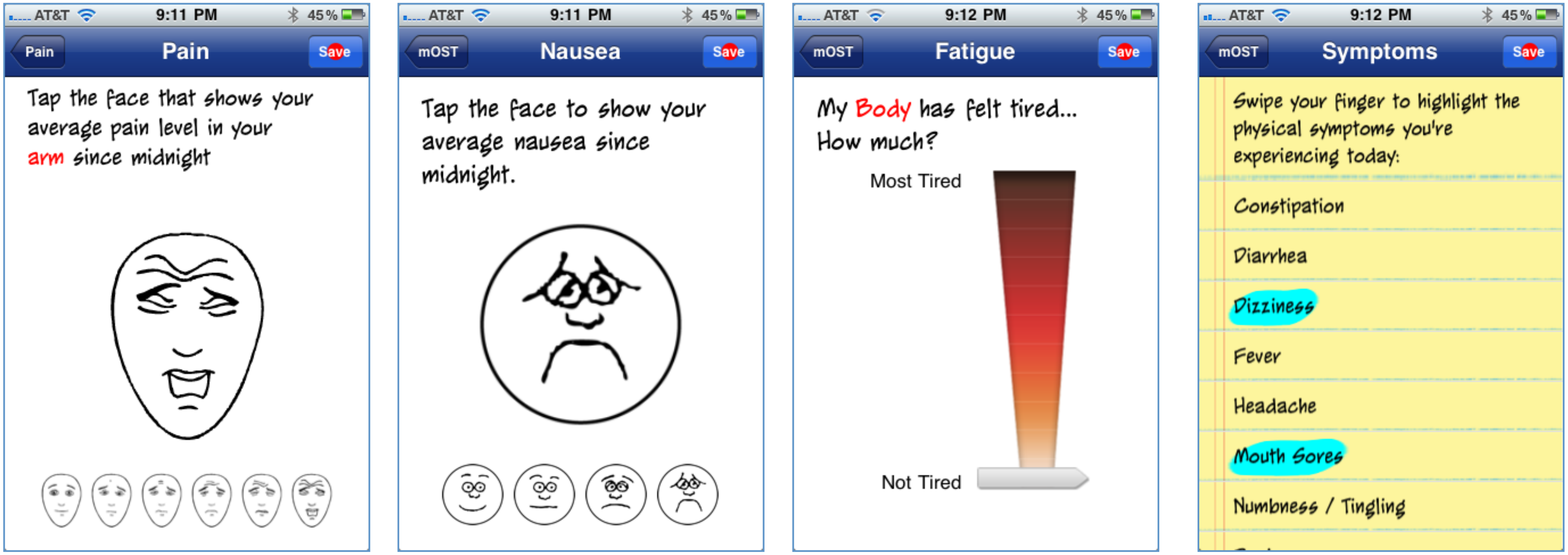

Mobile Oncology Symptom Tracker (mOST): Selected Screen Shots

From left to right:

- Pain ratings with the Faces Pain Scale-Revised (FPS-R)
- Nausea ratings with the Pediatric Nausea Assessment Tool (PeNAT)
- Physical fatigue ratings with a single item from the Fatigue Scale Adolescent
- Checklist for the occurrence of additional symptoms
